# Effectiveness of a fluid chart in outpatient management of suspected dengue fever: A pilot study

**DOI:** 10.1371/journal.pone.0183544

**Published:** 2017-10-04

**Authors:** Nazrila Hairin Nasir, Mohazmi Mohamad, Lucy Chai See Lum, Chirk Jenn Ng

**Affiliations:** 1 Department of Primary Care Medicine, Faculty of Medicine, University of Malaya, Kuala Lumpur, Malaysia; 2 Department of Paediatrics, Faculty of Medicine, University of Malaya, Kuala Lumpur, Malaysia; Institute of Tropical Medicine (NEKKEN), Nagasaki University, JAPAN

## Abstract

**Introduction:**

Dengue infection is the fastest spreading mosquito-borne viral disease in the world. One of the complications of dengue is dehydration which, if not carefully monitored and treated, may lead to shock, particularly in those with dengue haemorrhagic fever. WHO has recommended oral fluid intake of five glasses or more for adults who are suspected to have dengue fever. However, there have been no published studies looking at self-care intervention measures to improve oral fluid intake among patients suspected of dengue fever.

**Objective:**

To assess the feasibility and effectiveness of using a fluid chart to improve oral fluid intake in patients with suspected dengue fever in a primary care setting.

**Methods:**

This feasibility study used a randomized controlled study design. The data was collected over two months at a primary care clinic in a teaching hospital. The inclusion criteria were: age > 12 years, patients who were suspected to have dengue fever based on the assessment by the primary healthcare clinician, fever for > three days, and thrombocytopenia (platelets < 150 x 10^9^/L). Both groups received a dengue home care card. The intervention group received the fluid chart and a cup (200ml). Baseline clinical and laboratory data, 24-hour fluid recall (control group), and fluid chart were collected. The main outcomes were: hospitalization rates, intravenous fluid requirement and total oral fluid intake.

**Findings:**

Among the 138 participants who were included in the final analysis, there were fewer hospital admissions in the intervention group (n = 7, 10.0%) than the control group (n = 12, 17.6%) (p = 0.192). Similarly, fewer patients (n = 9, 12.9%) in the intervention group required intravenous fluid compared to the control group (n = 15, 22.1%), (p = 0.154). There was an increase in the amount of daily oral fluid intake in the intervention group (about 3,000 ml) compared to the control group (about 2,500 ml, p = 0.521). However, these differences did not reach statistical significance.

**Conclusion:**

This is a feasible and acceptable study to perform in a primary care setting. The fluid chart is a simple, inexpensive tool that may reduce hospitalization and intravenous fluid requirement in suspected dengue patients. A randomized controlled trial with larger sample size is needed to determine this conclusively.

**Trial registration:**

International Standard Randomized Controlled Trial Number (ISRCTN) Registry ISRCTN25394628 http://www.isrctn.com/ISRCTN25394628

## Introduction

Approximately 50 million dengue infections occur yearly, making it the fastest spreading mosquito-borne viral disease in the world[1, 2]. In 2002, dengue deaths amounted to 12,000 in South-East Asia, 4,000 in the Western Pacific and 2,000 in America[3]. Dengue has a wide spectrum of clinical presentations, ranging from a non-severe illness whereby outpatient treatment would suffice, to severe disease, hallmarked by plasma leakage requiring hospitalization. With increasing severity of plasma leakage, hypovolaemic shock ensues and the morbidity and mortality rises sharply. According to one study, the estimated cost of a non-fatal ambulatory case was US$ 514, and the cost for a non-fatal hospitalized case was US$ 1,491[4]. Thus, a hospitalized dengue case cost on average three times more than an ambulatory case. Moreover, dengue has considerable impact on the quality of life (QoL). One study found that all dengue patients experienced a drastic fall in their quality of life starting from the onset of symptoms[5].

Furthermore, dengue patients are more likely to be susceptible to dehydration caused by the high fever, vomiting, diarrhea and associated anorexia, which, if not carefully monitored and treated, may lead to shock, particularly in those with dengue haemorrhagic fever.[6]. Intravenous fluid is part of the therapy given for suspected dengue patients with dehydration. However, fluid overload frequently occurs during the management of dengue patients and is a risk which is not present when fluid is consumed orally. Despite the fact that oral rehydration therapy is incorporated in the standard outpatient management of dengue, the discussion on the use of such therapy for dengue is scarce. The study conducted in Nicaragua was the first study to examine the effect of fluid intake at home for dengue cases[6]. The results from this study showed that an oral fluid intake of 5 glasses per day decreases the risk for hospitalization of dengue patients[6]. In fact, based on this study, WHO had revised their dengue guidelines in 2009 and it includes the recommendation of oral fluid intake of five glasses or more for adults who are suspected to have dengue fever (e.g. milk, fruit juice, oral rehydration solution, barley, rice water or coconut water) [1]. The authors also postulated that oral fluid intake may have an effect on the severity of dengue[6]. However, causality cannot be confirmed due to the observational nature of this study. Thus, the authors of this study suggested prospective interventional studies be carried out to definitively demonstrate this effect[6]. It is important to confirm this because there are many advantages in encouraging oral fluid intake for suspected dengue patients, in terms of averting the risk of fluid overload with all its complications, cost savings in preventing intravenous fluid and the need for admission and possibly better quality of life when admission is avoided.

Since the study conducted in Nicaragua, other studies have been conducted with regards to oral fluid therapy in dengue. A non-randomized controlled study was conducted in Taiwan comparing the effects of oral rehydration with intravenous fluid replacement in adult patients with non-shock dengue haemorrhagic fever[7]. The study found that both interventions were equally effective. However, the sample size of this study was small and the participants were recruited from the hospital in-patient. There was one interventional, randomized trial on syndromic approaches for undifferentiated fever and dengue done at the primary health care setting in Vietnam[8]. However, although this study was conducted in primary care, it did not focus on oral fluid intake in dengue patients.

Most importantly, there have been no published studies looking at self-care intervention measures to improve oral fluid intake among patients suspected of dengue fever. Should there be an association between increased oral fluid intake and a decreased risk of hospitalization, it would be beneficial to explore practical methods of improving the oral fluid intake in patients with dengue fever. The study should be conducted in the primary care setting where there is immense potential for early intervention. Thus, this pilot randomized controlled study aimed to assess the feasibility of using a fluid chart to improve oral fluid intake in patients with suspected dengue fever in a primary care setting.

## Materials and methods

This was an unblinded, parallel-group study with balanced randomization (1:1 ratio) for the two groups of patients. The study was conducted from 1^st^ June 2010 – 30^th^ July 2010 (including recruitment and follow up) in a single centre i.e. Primary Health Care Clinic in the University Malaya Medical Centre (UMMC). This study was part of a Master dissertation.

Although the study was completed in 2010, there was a delay by the author in writing it up as a full manuscript. Ethical approval was obtained from the UMMC Medical Ethics Committee. Written informed consent was obtained from the participants. For child participants, written consent was obtained from the participants’ parents or guardian. This method was approved by the ethics committee.

The inclusion criteria were: Age > 12 years, patients who were suspected to have dengue fever based on the assessment by the primary healthcare clinician, history of fever for three days or more, and thrombocytopenia (Platelets < 150 x 10^9^/L)[9–11]. The exclusion criteria were: Patients with any type of current or active malignancy, human Immunodeficiency virus (HIV) and other immunodeficiency states due to complicated and multi-factorial aetiologies. Patients who were not given a follow-up appointment to review the clinical progression and to repeat the full blood count test were also excluded. This was to ensure that the patients enrolled in this study were similar to the target population, which were suspected dengue patients who were being followed up for outpatient monitoring. Similarly, those who were assessed on the first outpatient clinic visit and found to require hospital admission were excluded from this study.

**Fig 1:**
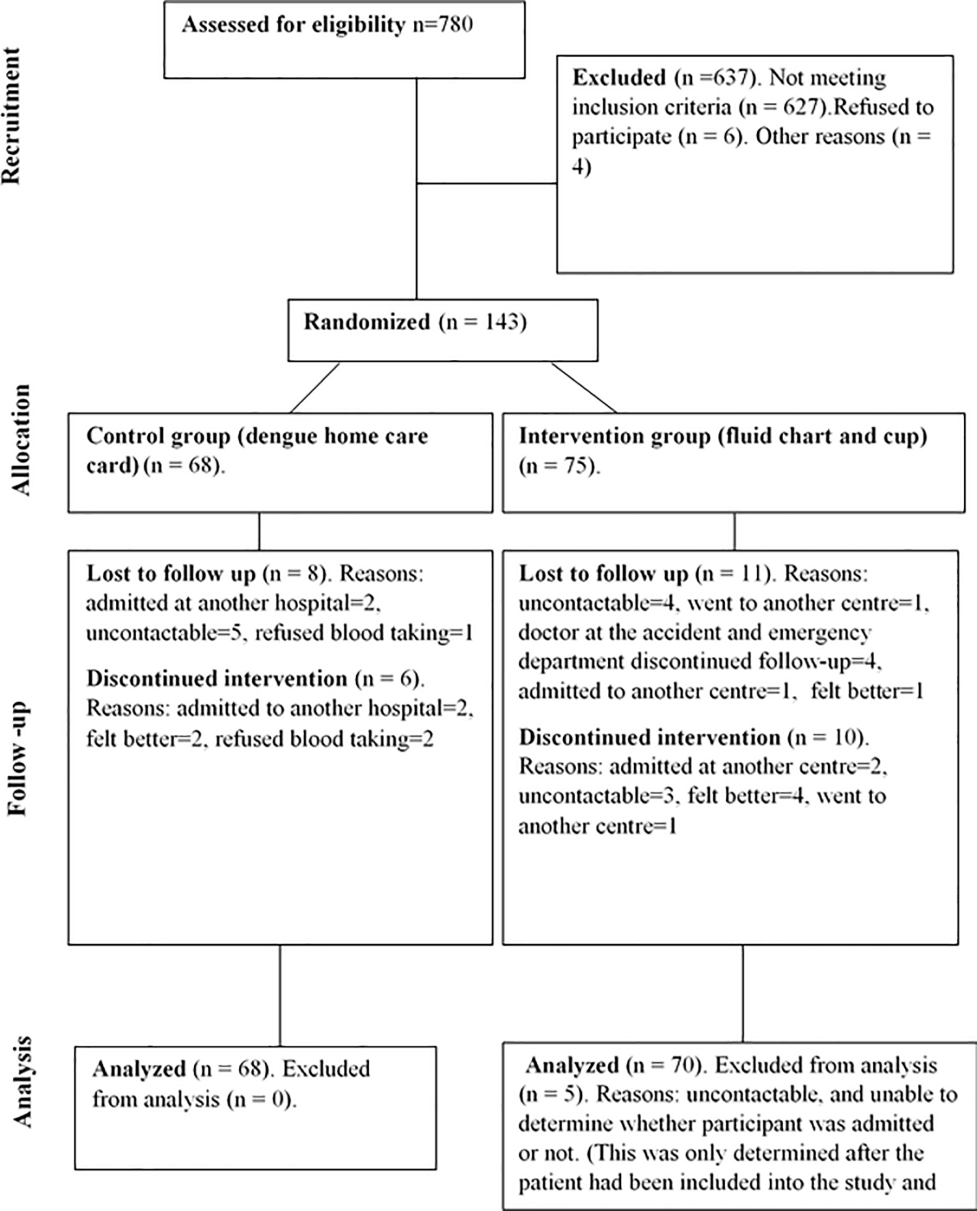
The CONSORT diagram.

**Fig 2:**
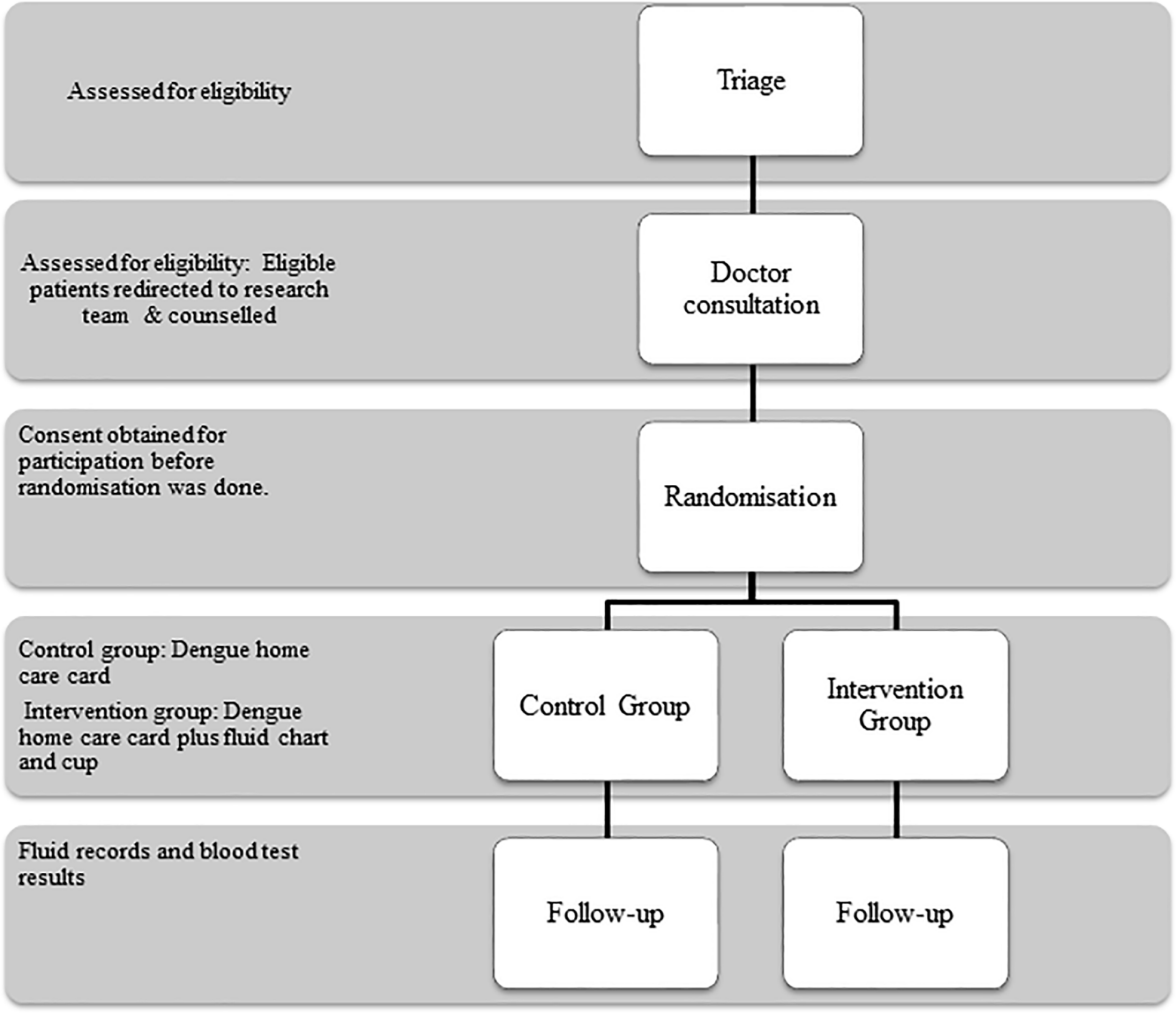
Flow of participants and trial process.

The process of recruitment and follow-up of the participants is shown in Fig 1 while the data collection process of the trial is shown in Fig 2.

All patients presenting to the clinic with a history of fever for 3 days duration or more were identified by the triage counter nurses. The phases of dengue fever were based on the day of illness: febrile phase—day 1 till day 3 of illness; critical phase—day 4 to day 6 of illness; recovery phase—day 7 of illness onwards. It was standard practice for the clinic that such patients would have a full blood count test done mostly before the patient’s consultation with their doctors. Online results of clinical investigations of patients were traced and those fulfilling the inclusion criteria were invited to participate in the study. The initial assessment of the patients and the subsequent follow-up consultations were conducted by the respective doctors working at the clinic. After the consultation, the researcher and trained research assistants obtained consent from the patient before they were randomized into either the control or intervention group. Simple randomization was done, with a 1:1 allocation ratio, using the table of random numbers. The table of random numbers was a standard table obtained from a statistics textbook. The researchers referred directly to the table during the randomization process (no opaque envelope was used).

All patients recruited into the study, regardless of randomization group, were given the standard care in the form of the dengue home care card. The information on this card was read out to all participants. The control group would be asked to recall their twenty four hour fluid intake, and details on what type of fluids was consumed. This information was obtained during recruitment and each follow-up.

The intervention group participants were given a plastic cup of uniform size (200ml), along with a twenty-four hour fluid chart. These patients were instructed to use the cup for drinking any form of fluid they wished to consume for the duration of their participation in the study. A fluid chart was used to record the detailed fluid intake (S1 File) and verbal instructions were given on how to fill in the fluid chart. Should the patient receive intravenous fluid during the following twenty-four hours either from a clinic or from the emergency department, they would record in the fluid chart given the time and amount of bottles received. In addition, demographic, clinical and laboratory data were collected.

All the patients recruited into the study were followed up until they had improved clinically and/or biochemically as determined by their doctors. This means that the duration of follow up varies between different participants. Data collection involved 24-hour fluid recall for the control participants, and the completed fluid charts from the intervention group. The research team would give out a new fluid chart if another appointment had been given to the participant. For both groups, the following data were recorded for each subsequent clinic follow-up: blood investigations (haemoglobin, haematocrit levels, total white cell count, platelets, and dengue serology) and temperature readings. The participants were managed by the respective doctors. As long as some data were collected from the participant, they would be included in the analysis. Only those participants who defaulted directly after recruitment into the study (meaning that they only came to clinic during recruitment) were excluded from the analysis as no clinical and oral fluid consumption data could be collected from them.

For those who defaulted their follow-up with the research team, an attempt to contact the participant via telephone was made, with a maximum of three attempts. The reasons for defaulting follow-up were elicited and if the patient was admitted, details regarding the admission were obtained.

The only published study on oral hydration in dengue patients [7] did not state their estimated sample size. For this pilot study the sample size was time driven, and the pre-specified time limit for data collection was two months. The minimum recruited number of participants required for each study group was 30 patients, giving a minimum number of 60 patients in total. However, the research team strived to recruit as many patients as possible during those two months. This is to determine the actual treatment effect size, and the participant recruitment and retention rates to be used in the sample size calculation for the main evaluation trial. The data for this pilot study were expressed either as the mean ± standard deviation or as frequencies and proportions. Differences in data between the control and intervention group were analyzed using the Student’s t test for continuous variables. The chi-squared test (χ^2^ test) or the Fisher’s exact test was used for categorical variables, whichever was appropriate. A p value < 0.05 was considered as statistically significant. The statistical software utilized for the analysis was SPSS version 16.0 (SPSS Inc. Cary, NC). The main outcomes were hospitalization rates, need for intravenous fluid treatment and amount of oral fluid intake.

## Results

The baseline socio demographic and clinical profiles were similar in both the intervention and control groups (Table 1). Most participants were in the critical phase of dengue infection, with slightly more participants in the recovery phase in the intervention group. However, there were more participants who had a documented body temperature > 38°C in the control group than the intervention group. More than half of the participants were confirmed to have dengue fever.

**Table 1:** Baseline sociodemographic and clinical data of the participants.

Sociodemography	Control (n = 68)	Intervention (n = 75)	P value
Age, mean (SD)	29.0 (±10.1)	29.1 (±13.8)	0.964
Gender: Male, n (%)	42 (61.8%)	48 (64.0%)	0.918
Ethnicity, n (%)			
Malay	30 (44.1%)	27 (36.0%)	0.036
Chinese	8 (11.8%)	23 (30.7%)	
Indian	15 (22.1%)	18 (24.0%)	
Other Malaysians	2 (2.9%)	1 (1.3%)	
Other nationalities	13 (19.1%)	6 (8.0%)	
**Clinical profile**			
BMI, mean (SD)	22.5 (± 4.2)	22.1 (± 4.5)	0.553
*Dengue phase*, *n (%)*			
Febrile	6 (8.8%)	4 (5.3%)	0.24
Critical	49 (71.1%)	48 (64.0%)	
Recovery	13 (19.1%)	23 (30.7%)	
Body temperature >38°C, n (%)	13 (20.0%)	2 (2.7%)	0.051
Haematocrit, mean (SD)	0.44 (± 0.04)	0.43 (± 0.04)	0.263
Total white blood cell count, mean (SD)	3.9 (± 1.8)	3.9 (± 1.6)	0.882
*Platelet*			
Platelet, mean (SD)	110 (± 27)	113 (± 25)	0.522
Platelet ≤ 100,000 cells/cumm, n (%)	20 (29.4%)	21 (28.0%)	0.999
Positive IgM, n (%)	37 (58.7%)	40 (61.5%)	0.886
Positive IgG*, n (%)	10 (15.9%)	10 (15.6%)	0.997
*Diagnosis*, *n (%)*			
Dengue fever	37 (54.4%)	40 (53.3%)	0.689
Probable dengue fever*	8 (11.8%)*	8 (10.7%)*	
Other febrile illness	18 (26.5%)	17 (22.7%)	
Undetermined	5 (7.4%)	10 (13.3%)	

*There were 10 participants from both study groups who had a positive IgG SD capture ELISA and a negative IgM SD capture ELISA result during the first sample for dengue serology. However, for 2 of these participants, the IgM SD capture ELISA became positive during the second dengue serology sample. Thus, 2 out of 10 of these participants had serological evidence of acute dengue infection, and 8 participants had probable dengue infection. Furthermore, the dengue serology sample was not taken for 5 participants (7.4%) in the control group and 10 participants (13.3%) in the intervention group. Thus, the diagnosis for these patients could not be determined.

The pilot study found a positive trend towards a reduction in the need for hospitalization and intravenous fluid treatment for the participants in the intervention group compared with those in the control group (Table 2). Among the 138 participants who were included in the final analysis, there were fewer hospital admissions in the intervention group (n = 7, 10.0%) than the control group (n = 12, 17.6%) (p = 0.192). Similarly, fewer patients in the intervention group (n = 9, 12.9%) required intravenous fluid compared to the control group (n = 15, 22.1%) (p = 0.154).

**Table 2:** Comparison of the clinical outcomes between the intervention and control groups.

Clinical outcomes	Control (n = 68)	Intervention (n = 75)	P value	OR / mean difference	95% CI
Hospitalization, n (%)	12 (17.6%)	7 (10.0%)	P = 0.192	0.52	(0.191 to 1.41)
Requiring intravenous fluid, n (%)	15 (22.1%)	9 (12.9%)	P = 0.154	0.52	(0.211 to 1.29)
**Haematocrit**					
Mean peak haematocrit, mean (SD)	0.45 (±0.03)	0.44 (±0.04)	P = 0.310	0.01	(-0.01 to 0.02)
Cut off points reached, n (%)	44 (66.7%)	36 (52.9%)	P = 0.105	0.56	(0.28 to 1.13)
Haematocrit fluctuation ≥ 20%, n (%)	1 (1.5%)	3 (4.4%)	P = 0.619	3.00	(0.30 to 29.59)
Difference between clinic visit 2 and clinic visit 1, mean (SD)	-0.02 (±0.02)	-0.02 (±0.02)	P = 0.937	0.00	(-0.01 to 0.07)
**Platelet**					
Mean nadir platelet count (SD) (intervention, n = 68, control, n = 66)	95 (±31)	101 (±29)	P = 0.286	-6.89	(-23.31 to 9.53)
Mean difference between clinic visit 2 and clinic visit 1 (SD) (intervention n = 67, control n = 64)	4 (±37)	11 (±55)	P = 0.408	-5.49	(-15.65 to 4.66)
Average daily fluid intake (ml)	2514.1 (±1138.9	3020.3 (±2033.9)	0.521	-506.2	(-1096.5 to 84.12)

The mean peak haematocrit, the difference in haematocrit values between the first two clinic visits, cut off points reached and the haematocrit fluctuation ≥ 20% for both groups were similar. The mean nadir platelet count and the mean difference in platelet levels between the first two clinic visits were also similar between both groups. The average daily fluid intake was higher in the intervention group (about 3,000 ml) compared to the control group (about 2,500 ml), but the difference did not reach statistical significance.

## Discussion

This is the first study that investigated the use of a fluid chart in the management of suspected dengue patients in a primary care setting. In this pilot study, the intervention group had 7.6% fewer hospital admission and 9.2% fewer participants requiring intravenous fluid therapy compared with the control group. The mean daily fluid intake in the intervention group was 500 ml more than that in study group but this increase was not statistically significant.

Although the differences observed did not reach statistical significance, there is a positive trend favouring the intervention group. Moreover, even a small reduction in hospital admission and the need for intravenous fluid for suspected dengue patients can potentially improve the quality of life of patients and cost savings to the economy of dengue endemic countries. The hospitalization rates of patients with suspected dengue fever in this study were 17.6% and 10% in the control and intervention groups respectively. These hospitalization rates were much lower than those during the preceding three months to the recruitment phase: 55.4% in March, 40% in April, and 63.4% in May 2010. [12] A prospective observational study in the same clinic in 2008 enrolled 214 patients aged 16 years and above with ≤ 72 hours of undifferentiated fever, 65% of whom had a laboratory confirmed diagnosis of dengue. Of the 140 patients with dengue, 11.4% developed plasma leakage and 37.1% required hospitalization. [13]

The marked reduction in hospitalization rates in both the control and intervention groups compared to previous observational studies might be due to the fact that the participants from both groups received instructions from the research team who had read out the advice written on the dengue home care card to each participant. The instruction included specific advice encouraging patients to increase their fluid intake to at least 5 cups of water a day.

This practice of reading out the advice written on the dengue home care card was not the standard practice of the doctors practicing in the study centre, neither is it a practice in most primary care clinics in Malaysia. The usual standard practice in primary care would be to give the dengue home care card to the patient with no verbal advice accompanying it, and it is up to the patient to read the card themselves. It is possible that reading out the dengue home care card became an active form of intervention in the control group, thus resulting in the control group participants to drink more fluids than they normally would had they received standard care. This could be a form of double intervention, and the difference in fluid intake between the two groups might be more pronounced if the dengue home care card was not read out to the participants, simulating the standard practice in primary care more closely. Thus, the true impact of the fluid chart on the fluid intake for suspected dengue patients might be underestimated in the current results. However, the intervention group seemed to have consistently maintained a higher mean fluid intake from day 1 to day 6 of the clinic consultations. Another possibility is that there might be other factors influencing the amount of fluid intake consumed by each participant, not just the fluid chart. However, this was not explored in this pilot study.

The fluid chart is an intervention tool which was designed to measure and document accurately the patient’s fluid intake which they should fill prospectively. By doing this, the patient’s awareness of his or her own oral fluid intake should be increased. This increased awareness could be the catalyst for a behavioural change, motivating the patients to increase their oral fluid intake. The improved oral fluid intake could translate into a reduction in morbidity. The reduction in morbidity is evidenced by the observable reduction seen in hospital admission and the reduced requirement for intravenous fluid therapy in the group that was given the fluid chart.

This study also found that the fluid chart was acceptable to the participants in the intervention group. There were only a few participants who did not fill the fluid chart prospectively, while the majority filled it as instructed. Moreover, almost all of the participants in the intervention group were able to understand the instructions given on how to fill it correctly. It could be concluded from this pilot study that the fluid charts were feasible to be used in the primary care setting.

The drop-out rates in the pilot study was 11.8% in the control group and 14.7% in the intervention group. This is much lower than the default rate of the general out-patient clinic at UMMC, which was found to be 33.8% in a previous study [14]. The same study found that the default rate was 16.4% amongst patients who were suspected to have dengue infection [14]. Thus, there were good participant retention rates, attesting to the good acceptability of the intervention.

Thus, this pilot study showed that the experimental intervention tool in the form of the fluid chart was able to produce a positive effect on the measured outcome. A larger randomized controlled trial might be able to produce results which are statistically significant. Thus, based on the preliminary results obtained during this pilot study, the fluid chart as a complex intervention should be evaluated further using a larger randomized controlled trial.

Future studies should be conducted to examine the relationship between the amount of oral fluid intake and the clinical outcome of dengue patients in primary care. By doing so, this would provide better evidence in order to recommend how much fluids patients with suspected dengue fever should consume orally. Future studies measuring the amount of fluid intake required to deflect the necessity of intravenous fluid and admission would provide a more accurate basis to change the current standard primary care practice of giving general advice. Further research in this area would then provide evidence whether five glasses of fluids consumed orally per day for those suspected with dengue fever would prove to be sufficient, or otherwise.

In this study, the participants were chosen on the basis that they were clinically suspected to have dengue fever, which is how the majority of dengue patients are managed and treated in primary care. This makes the participants in this study comparable to suspected dengue patients treated at the primary care level in Malaysia specifically and primary care in an Asian setting, generally.

However, this pilot study had several limitations. Due to the time constraint during the data collection, the sample size was small. Nevertheless, based on the findings from this study, using hospital admissions as the outcome measure (intervention 10.0% vs control 17.6%), with a power of 80% and p = 0.05, the estimated sample size needed for each arm would be 348. The dengue home care card was also read out to all participants, despite this not being the standard practice. This possibly became an active form of intervention in the control group, and a ‘double intervention’ in the intervention group, thus underestimating the true impact of the fluid chart on the fluid intake of the study participants. The randomization process in this pilot study using the table of random numbers with no allocation concealment might lead to bias. Thus, a computer generated sequence with block randomization and varied block size should be applied. Allocation concealment methods should also be used to avoid bias. We did not include other clinically relevant parameters such as dengue severity, shock and bleeding, as outcomes measures. These variables should be measured in the definitive trial.

This study was carried out in 2010, a few months after the introduction of the revised dengue classification of 2009 [15] which had yet to be disseminated to all primary health care facilities in Malaysia. The 1997 WHO dengue case classification [16] was not applied in primary health care settings because of inherent difficulties as reviewed by Bandyopadhyay et al, 2006 [17]. In the absence of clear guidelines for admission, the need for admission was left to the clinical judgment of the clinician managing the patient in the primary care setting. Due to a limited number of available beds in the infectious disease wards, it was an established practice for primary care clinicians to discuss with their colleagues in the Medical Department before patients were allowed to be admitted. This gate-keeping practice could reduce the number of unnecessary hospitalizations. It could be possible that a number of patients who were admitted would be classified as Dengue Fever (DF) according to the 1997 WHO dengue case classification [16]. However, this would be in keeping with the observations of a multicentre study that recorded 52% of DF patients (447 out of 861) needing intermediate interventions, including admission for intravenous fluid therapy. [18]

## Conclusions

This pilot study has ascertained that it is a feasible and acceptable study to perform in a primary care setting. The fluid chart is a simple, inexpensive, acceptable intervention that may reduce hospitalization and the requirement for intravenous fluid treatment in suspected dengue patients. A randomized controlled trial with larger sample size is needed to determine this conclusively.

## Supporting information

S1 File Intervention tool: Fluid chart. (PDF)

S2 File Study protocol. (PDF)

S3 File CONSORT 2010 checklist_fluidchart. (DOC)

S4 File Cummulative fluid score data. (XLSX)

S5 File Cummulative results output. (ZIP)

S6 File Cummulative fluid score data 20170322. (ZIP)
