# Supplementary material for: Effectiveness of a fluid chart in outpatient management of suspected dengue fever: A pilot study
Source: PLoS One. 2017 Oct 4;12(10):e0183544. doi: 10.1371/journal.pone.0183544 (PMC5627892; doi:10.1371/journal.pone.0183544)
Supplement: S1 File — (PDF) [file pone.0183544.s001.pdf]

S1 Appendix: Intervention tool: Fluid chart

Name / Nama:

Day and date / Hari serta tarikh:

How many days since the fever started / sudah berapa hari demam:

Time/masa	Number of cups that you drank /bilangan cawan yang diminum	Types of drink /jenis minuman	Number of fluid bottles received (drip): at clinic or A+E/bilangan botol drip yang diterima
**Morning / Pagi**			
8.00am			
9.00am			
10.00am			
11.00am			
12.00pm			
**Afternoon / Petang**			
1.00pm			
2.00pm			
3.00pm			
4.00pm			
5.00pm			
6.00pm			
**Night / Malam**			
7.00pm			
8.00pm			
9.00pm			
10.00pm			
11.00pm			
12.00am			
1.00am			
2.00am			
3.00am			
4.00am			
5.00am			
**Morning / Pagi**			
6.00am			
7.00am			
8.00am			
