# Supplementary material for: Effectiveness of a fluid chart in outpatient management of suspected dengue fever: A pilot study
Source: PLoS One. 2017 Oct 4;12(10):e0183544. doi: 10.1371/journal.pone.0183544 (PMC5627892; doi:10.1371/journal.pone.0183544)
Supplement: S2 File — (PDF) [file pone.0183544.s002.pdf]

Study Protocol: The effectiveness of a fluid chart in the outpatient management of suspected dengue cases: A pilot study

Background

Amongst all the mosquito-borne viral diseases in existence, dengue's spread worldwide is the swiftest. Its incidence has increased 30-fold over the last 50 years encompassing new geographic borders. Approximately 50 million dengue infections occur yearly. Those who are especially at risk are the 2.5 billion people living in dengue endemic countries, which encompass more than 100 countries in Africa, the Americas, the Eastern Mediterranean, South-east Asia and Western Pacific[1 2]. This constitutes two fifths of the world's total population.

From this figure, an estimated 1.8 billion (>70%) of those at risk are living in countries whom are member states for the WHO South-East Asia Region and Western Pacific Region [1]. According to the data compiled from the WHO registration system, there were 12,000 deaths in South-East Asia, 4,000 deaths in the Western Pacific and 2,000 deaths in America which were due to dengue during the year 2002[3].

Malaysia constitutes one of the WHO Western Pacific Region member state. Dengue was first documented in Malaysia in 1902, and it was made reportable during the year 1971[4]. The first outbreak of dengue haemorrhagic fever (DHF) occurred in 1962. Subsequently, 4-yearly outbreaks ensued until the year 1992 when dengue became endemic. Since then, frequent outbreaks happen yearly[5]. Malaysia's reported incidence for dengue has been consistently high, with an average of 125 to 167.76 per 100,000 people annually from 2002 to 2008 [6]. The total number of reported cases fluctuate from approximately 27,000 in 1998, 40,000 in 2005 to 24,634 cases in 2007 [6]. Notified cases from urban areas formed the majority, ranging from 62.0% - 86.7%[4 5].

There was also a shift of age-groups of those affected. Children were mainly affected initially until the 1982 epidemic, when young adults became the majority [7]. The mortality rate for 2008 was 0.02 per 100,000 people. The total number of dengue related deaths was 67 during 2006, rising to 82 deaths during 2007[6]. For 2009, the WHO report until 15th of June 2009 found that there were 163 dengue related deaths in the Western Pacific region. From this figure, Malaysia was the second highest contributor of dengue related deaths at 33.1 deaths after Philippines which had 38 deaths[8].

Dengue has a wide array of clinical presentations, ranging from a self-limiting, non-severe illness to severe disease, hallmarked by plasma leakage with or without haemorrhage. The epidemiology of dengue is also evolving. During 1962-1974, the initially recognised features were of sudden onset high-grade fever coupled with non-specific constitutional symptoms such as headache, retro-orbital pain, myalgia, vomiting, abdominal pain and a maculopapular rash[9 10]. With time, clinicians began to see rare, severe manifestations of

dengue encompassing cardiomyopathy, encephalopathy, encephalitis, fulminant hepatitis and renal involvement [9-12]. With increasing severity and hypovolaemic shock, the morbidity and mortality rises sharply. Since the majority of patients are those from the young adult age group, deaths occurring amongst children and the young adults represent an enormous loss of resources to the country and society as a whole.

Coupled with the rising morbidity and mortality is the significant economic and social stress. This is incurred on the affected individual, his or her household, and on the healthcare systems of nations suffering from epidemic or endemic dengue. According to one study assessing the cost of dengue in eight countries, the estimated cost of a non-fatal ambulatory case was US$ 514, whereas the approximate cost for a non-fatal hospitalized case was US$ 1,491[13]. This means that a hospitalized dengue case cost on average three times more than an ambulatory case [1 13]. The overall cost of officially reported dengue cases is estimated to be US$ 440 million[1]. This included the average annual number of officially reported cases for those eight countries[1]. However, this estimation did not include the underreporting of cases, the cost of dengue surveillance and vector control programmes. Thus, each treated episode of dengue results in significant costs, not only to the health sector but also to the overall economy of various nations worldwide.

The major contributors to the nations' workforce are young adults, who would be unable to work during the illness. Thus, the working days lost also causes a large drain of the nations' resources. Dengue affects other household members who have to care for the dengue patient, and this has to be economically accounted for. Thus, one episode of dengue illness causes on average 14.8 lost days for an ambulatory patient and 18.9 days for a hospitalized patient[1].

However, cost is not the only consideration. Dengue has considerable impact on the quality of life (QoL) of those afflicted with it. The first study assessing this found that all patients experienced a drastic fall in their quality of life starting from the onset of symptoms[14]. The nadir of quality of life (40% of healthy status) was experienced between the third and seventh days of illness. However, the quality of life in ambulatory patients also recovered sooner than hospitalized patients [14].

Furthermore, the duration of impaired quality of life for hospitalized patients of 13 days was significantly longer than the ambulatory patients (9 days). The duration of fever for ambulatory patients was an average of 5 days and for those hospitalized patients was 7 days. When this is being compared with the mean duration for impaired quality of life, the duration of impaired quality of life was longer than the duration of fever[14]. Thus, not only do patients with dengue have a significant reduction in their quality of life during the infection, the effects linger on for longer than the actual duration of fever.

In an effort to aid the management of dengue cases, to curb the spread of dengue and curtail its negative impact, the WHO formulated guidelines for the diagnosis, treatment, prevention and control of dengue. This was first devised in 1974, with subsequent revisions in 1986, 1994 and 1997. The 1997 WHO classification system categorized patients into dengue fever, dengue haemorrhagic fever (DHF) and dengue shock syndrome (DSS) [15]. However,

universally, clinicians found significant difficulties trying to use this classification system [16-18]. Thus, the WHO have revised their guidelines in 2009, with a new, simplified classification system of dengue with or without warning signs and severe dengue[1].

In addition to the new classification system, the WHO guidelines 2009 contains an action plan for ambulatory patient management. Primary health care providers are to encourage oral fluid intake for suspected dengue patients. The recommended fluid types are oral rehydration solution (ORS), fruit juice and other fluids containing electrolytes and sugar. The WHO included a statement that adequate oral fluid intake might reduce the number of hospitalizations[1]. They also devised a home care card for ambulatory patients suspected of dengue undergoing regular monitoring and follow-up with a primary health care facility.

The advice stipulated on the home care card is as follows: patients are advised to have adequate fluid intake of more than 5 glasses for average-sized adults or accordingly in children. The types of oral fluids encouraged are milk, fruit juice, isotonic electrolyte solution (ORS), along with barley or rice water[1]. There is a note cautioning that plain water alone may cause electrolyte imbalance.

General advice such as adequate bed rest, paracetamol usage, tepid sponging and identifying and eliminating mosquito breeding places in and around the home is included on the card. The home care card also advices patients to avoid non-steroidal anti-inflammatory agents (NSAIDS) and steroids, and informs them that antibiotics are not necessary. The home care card also states the warning signs of dengue, and a table for laboratory results monitoring which includes the haematocrit, white cell count and platelet count values[1]. The effectiveness of this home care card has yet to be assessed.

The new recommendations for patients to drink 5 or more glasses along with the types of fluid encouraged were made based on a single study done in Nicaragua[19]. This study is the first of its kind looking into the possibility that increased oral fluid intake decreases the risk for hospitalization of dengue patients [19]. Prior to this, there have been no published studies evaluating the effectiveness and importance of oral fluid intake in improving the morbidity of patients with dengue fever. This is ironic, as encouraging oral fluid intake is a standard outpatient management of dengue patients even prior to the revision of the WHO guidelines.

Since this Nicaraguan study, another study had been done to compare the effects of oral rehydration and intravenous fluid replacement in adult patients with non-shock dengue haemorrhagic fever[20]. The findings of this study were suggestive that oral hydration may be equivalent in its effectiveness as intravenous hydration for volume replacement[20]. This study adds evidence to support the effectiveness of oral fluid intake in reducing the significant morbidity related to dengue infection. The findings of both these studies infer that it could be possible to prevent hospitalization and reduce the need for intravenous fluid therapy by encouraging dengue patients to increase their oral fluid intake. This measure could have enormous implications in terms of cost reductions in healthcare and economic savings as a whole.

Although there has been some evidence that encouraging oral fluid intake might decrease the morbidity of dengue infections, it is still uncertain whether oral fluid intake exerts a protective effect against the progression from non-severe to severe dengue [19]. It is still unknown whether increasing oral fluid intake would alter the progression of dengue in terms of the degree of haemoconcentration and the fall in platelets.

Both of these parameters are routinely used for monitoring of both outpatients and hospitalized suspected dengue patients. Primary healthcare providers monitor these parameters very closely. Coupled with the clinical condition, primary physicians determine the necessity for hospital admission. Moreover, both these parameters constitute part of the warning signs mentioned in the WHO 2009 guidelines[1]. However, it is still unknown whether increasing oral fluid intake in dengue patients will improve the mortality of dengue by lessening its progression or severity.

From the two studies looking at the effects of oral fluid intake, more questions arise. Should there be an association between oral fluid intake and the risk of hospitalization for dengue patients, an accurate estimation of the amount of fluid needed daily to achieve this effect is not known. The Nicaragua study collected data by retrospectively asking the patients how many glasses of fluid was ingested during the previous 24 hours[19]. The findings are subject to recall bias. Moreover, there is a great variation in size of the glasses and cups used by the general population. Therefore an accurate estimate of the amount of fluid ingested daily could not be obtained.

The Taiwan study did not collect any data on the amount nor the type of oral fluids consumed by the patients in their oral hydration group[20]. The only study that had some data on the types of fluids consumed by their patients was the study done in Nicaragua[19]. Thus, little is known on the kinds of fluids that would be ideal for oral hydration in dengue patients. Such knowledge would greatly aid the primary healthcare members as well as other clinicians in providing accurate, evidence based advice regarding the quantity and types of fluids for dengue patients to consume in order to avoid hospitalization.

Moreover, most of the published dengue studies thus far were conducted in an inpatient setting, while some were conducted jointly between primary and secondary care. Unfortunately, very little studies have been done purely in the outpatient setting. This is a paradox, since primary healthcare centres are the first point of contact for most suspected dengue cases. The primary healthcare clinicians manage most of the non-severe dengue, monitoring closely for warning signs and refer appropriately. So far, there was one interventional, randomised trial done at the primary health care setting in Vietnam[21]. This study looked at interventions to improve the diagnosis and treatment of syndromic approaches for undifferentiated fever and dengue[21].

Most importantly, there have been no published studies looking at strategies and interventions to improve oral fluid intake amongst hospitalized or ambulatory dengue patients. Should there be an association between increased oral fluid intake and a decreased risk of hospitalization, it would be extremely beneficial to explore practical methods of improving the oral fluid intake in dengue patients. This would best be

conducted in a primary care setting as most of the dengue patients will initially present to the front liners. The WHO 2009 home care card for dengue patients that was recently introduced seems very useful. However, no studies so far have assessed the effectiveness of this home care card in improving the morbidity and mortality of dengue patients.

Complex interventions are defined as interventions that contain several interacting components[22]. These components may act independently and inter-dependently, and could include behaviours, parameters of behaviours (like frequency and timing), and methods of organising and delivering those behaviours (for example practitioner type, setting and location). It is usually not easy to define the 'active ingredients' of a complex intervention with precision. Complex interventions can be directed at the level of individual patient care, organisational or service level, health professionals or at the population level[23].

The fluid chart which is an experimental intervention tool that was developed for this pilot study, is a form of complex intervention. In this study, there are three components that interact independently, and with each other. The main components are as follows:

a) Fluid chart and cup
b) Healthcare provider administering the tool
c) Patient understanding and adherence to the tool

The literature review, designing and developing the fluid chart and planning for this pilot study was all part of the theory and modelling process. This was followed by the pilot study which is an exploratory trial in Phase II of the MRC framework. This exploratory trial tests out the experimental intervention and the process of evaluation. This pilot study also generates and determines the trend of the preliminary results crucial to calculate the sample size for the definitive randomised controlled trial.

The first point of medical care for febrile patients living in any community at large will usually be the primary healthcare centres. In addition to this, patients tend to consult the primary healthcare providers early when they have fever[25]. However, the clinical features of early dengue fever are very similar to that of other febrile illnesses, making the distinction between the two fraught with difficulties. Many studies have attempted to identify differentiating clinical and laboratory features between dengue and other febrile illnesses[26-29]. Some studies have proposed the usage of decision tree algorithms and diagnostic models to aid primary care clinicians to identify probable dengue cases early[26-29]. However, none of these diagnostic models or decision algorithms have been validated or widely used in other countries.

Another added barrier to early diagnosis of dengue fever is that to confirm a case of acute dengue infection, the serologic test results are only obtained after several days up to one week. This is because detection of newly formed antibodies (IgM) would not be possible until after viraemia ends or after fever subsides[30] .The first detectable dengue IgM begins to appear on day 4 to day 5 of illness[31]. Expensive laboratory tests yielding faster results are usually not available in resource-poor countries.

Due to the delay in confirmatory test results, those who require admission are hospitalized with a provisional diagnosis of suspected dengue fever. Ideally, only patients with confirmed dengue fever with warning signs or severe dengue should be admitted as they may develop severe manifestations and rapid deterioration. Those patients with other febrile illnesses could then be managed in a more cost effective and appropriate way. As stated earlier, hospital admission increases the healthcare costs by three fold when compared with an ambulatory patient managed at the primary healthcare clinics[1].

Due to the similarity in the early presentation between dengue fever and other febrile illnesses, and the reliance on dengue serology yielding results after one week for confirmatory diagnosis, most primary care clinicians are faced with diagnostic uncertainty[25]. The subsequent management is then based on a presumptive diagnosis of suspected dengue fever. However, in spite of these difficulties, it is undeniable that there exists a great potential for intervening early in the course of illness. By intervening early at the primary healthcare level, it could be possible to reduce hospitalization of patients by avoiding progression of the disease severity.

The definition for self-care is as follows: Self-care is the care taken by individuals towards their own health and well-being[32]. There has been a move for general practitioners and healthcare providers to promote higher levels of self-care amongst the members of the public. In the United Kingdom, the National Health Service (NHS) has developed an Expert Patient Programme, advocating self-care[33]. This approach of self-care can be applied to a multitude of areas and conditions.

There is currently a vast array of self-care support interventions. This includes information-giving, addressing motivations and barriers to change, teaching coping strategies, designing action plans, consistent monitoring, and engaging family and social support[34]. Since the patient takes an active part in their own management, this might improve their own outcome. Providing a home care card or a fluid chart as a self-care intervention for suspected dengue patients in primary care could potentially yield an improvement in outcome.

Thus, in summary, there is emerging evidence that increased oral fluid intake could reduce the risk of hospitalization for dengue patients, and that oral hydration might be as effective as intravenous fluid for volume replacement in adults with non-shock dengue[19 20]. There is also an immense potential for early intervention in primary care to improve the morbidity and mortality of dengue patients. This could possibly be achieved by using self-care intervention measures in the form of the home care card and the fluid chart.

However, there have been no published studies to date that have assessed the effectiveness of these interventions to increase the oral fluid intake and improve outcome in suspected dengue patients. Due to the diagnostic difficulty faced by primary healthcare clinicians assessing early presentation of febrile illness, most are managed based on a presumptive diagnosis of suspected dengue fever.

Therefore, this pilot study aims to explore the feasibility of conducting a randomised controlled trial in a primary healthcare setting to assess the effectiveness of a fluid chart in improving oral fluid intake of suspected dengue patients. This pilot study also aims to address four primary questions:

1. Whether it is feasible to assess the fluid chart to improve oral fluid intake in suspected dengue patients in a primary care setting with a randomized control design
2. Whether the fluid chart is an acceptable intervention for suspected dengue patients in a primary care setting
3. To determine the trend of the preliminary results in order to estimate the size of the treatment effect of the fluid chart as an experimental intervention
4. To determine the sample size of the main evaluation trial by generating preliminary results for the sample size calculation

Research question

Is it feasible to evaluate the effectiveness of a fluid chart to improve the oral fluid intake of suspected dengue patients in a primary care setting?

Aim

To assess the feasibility and acceptability of conducting a randomised controlled trial to evaluate the effectiveness of a fluid chart compared with 24 hour fluid recall to improve the oral fluid intake of suspected dengue patients in a primary care setting

Study objectives

1. To determine the feasibility of the designed research process in terms of the following:
    a. Recruitment/retention of participants
    b. Randomisation of the participants.
    c. Logistics – manpower, resources
2. To determine the feasibility and acceptability of the designed intervention in terms of the following:
    a. Usage of the cups – as a standardized measure of fluid intake
    b. Usage of the fluid charts – as a tool for behaviour change
    c. The implementation process of the intervention
3. To determine the trend of the preliminary results in order to:
    a. To estimate the treatment effect size of the experimental intervention
    b. To calculate the sample size for the main evaluation randomized controlled trial.
4. To refine the research process and intervention design for the main evaluation randomized controlled trial.

Outcomes

1. The issues and identified problems arising from the feasibility/piloting process
2. The feasibility and acceptability of the designed intervention
3. The trend of the preliminary results in terms of the following:
    a. Hospitalization rates
    b. The need for intravenous fluid treatment
    c. The severity of haemoconcentration
    d. The severity of platelet drop
    e. The amount and type of oral fluid intake
4. The sample size for the main evaluation trial

Methods

As this is a feasibility study, a detailed outline regarding the study design and research process will be mapped out in this chapter. The study population will be defined, along with the method of recruitment and randomization. The intervention method employed in the intervention group, the control group and in both groups will be explained. The processes for data collection, follow-up, blood tests and defaulter tracing will be clarified. The preliminary outcomes as well as the preliminary endpoints will require further explanation. The estimated sample size, the statistical analysis and the terminologies, definitions and assumptions used for this pilot study will be illuminated.

Study design

This is a single centre, unblinded, parallel-group study with balanced randomisation (1:1 ratio) for the two groups of patients.

Study setting

The study is conducted from a single centre, which is the primary health care clinic situated within the grounds of the University Malaya Medical Centre (UMMC). This clinic is part of the University of Malaya Medical Centre, which is a tertiary education centre and a semi-government, partly private paying institution. Therefore, those who are not employed by the government have to pay for their blood tests and the medication.

This primary healthcare clinic has 30 consultation rooms and two treatment rooms situated on two floors of a three storey building. There are generally 20-30 doctors attending to the patients in any given day. This clinic is situated in an urban setting, catering for patients whom are mainly from Petaling Jaya, the Klang Valley and Kuala Lumpur area. Kuala Lumpur is the capital city of Malaysia. The clinic caters for the surrounding population whom are mainly working and from the lower to middle class.

Malaysia's population is of multi-ethnic background, with the main ethnicity being the Malays, the Chinese and the Indians. The climate of Malaysia is tropical, with two monsoon seasons in a year, and dengue is endemic in this country. During the three months prior to the recruitment phase, the estimated percentage of cases of suspected dengue fever whom were admitted from this clinic to the hospital were as follows: 55.4% were admitted in March, 40% in April, and 63.41% were admitted during May 2010.

Time frame of data collection:

The data will be collected from 1st June 2010 – 30th July 2010.

Ethical approval

Ethical approval will be obtained for the study from the Medical Ethics Committee for the University of Malaya Medical Centre.

Study population: Participants

Inclusion criteria

All patients with the following:

1) Patients who are suspected to have dengue fever from the history and examination findings by a primary healthcare clinician.
2) History of fever for three days or more.
3) Thrombocytopenia: Platelets equal to or less than 150 x 10^9/l.
   The platelet level of 150 x 10^9/L is chosen as the highest cut off point for thrombocytopenia because it is the lowest level of the normal range for platelets according to the University of Malaya Medical Centre's clinical laboratories. During the early febrile illness, the platelets and total white blood cell count are found to be lower among patients with dengue compared with patients with other febrile illness[26 48 49].
4) Age equal to or more than 12 years old. This is arbitrarily set to ensure that the patient concerned can understand instructions and fill in the fluid chart reliably and independently.

Exclusion criteria

Patients with any of the following:

1. Any type of current or active malignancy
2. Human Immunodeficiency virus (HIV)
3. Serious underlying medical conditions conferring immunodeficiency. Immunodefiency states such as HIV or serious medical conditions were excluded from the study as febrile illnesses in these patients are usually due to complicated and multifactorial aetiologies.
4. Patients who are not given a follow-up appointment to review the clinical progression and to repeat the full blood count test. This is to ensure that the patients enrolled in this study are similar to the target population, which are patients who are suspected to have dengue fever by a primary health care provider and are being followed up for outpatient monitoring.
5. Patients who are assessed on the first outpatient clinic visit and found to require hospital admission were excluded from this study.

Method of recruitment

Figure 2: Flow of participants and trial process

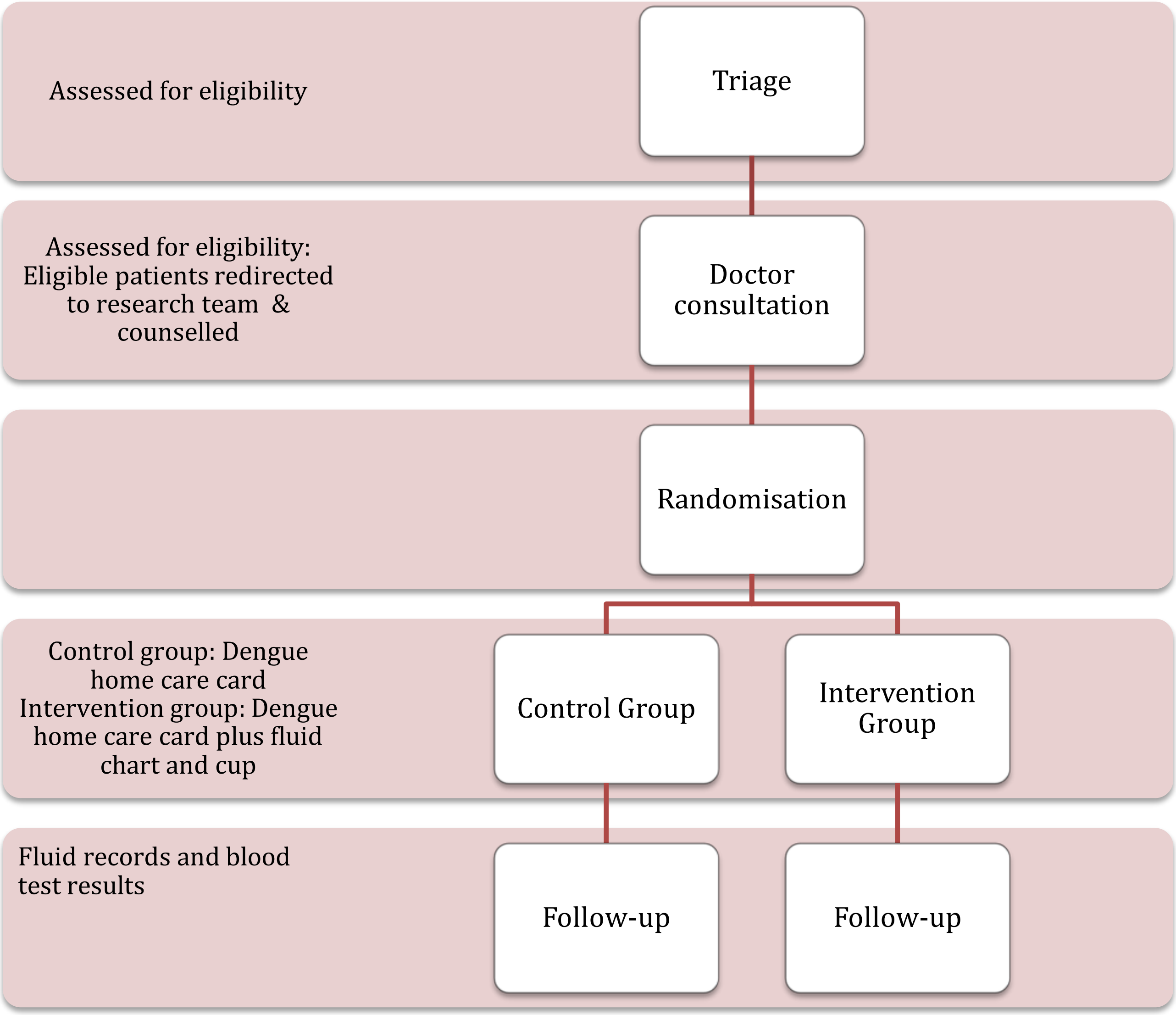

Screening

All the patients presenting to the clinic with a history of fever for 3 days duration or more will be identified by the triage counter nurses. The clerk at the registration desk who handles the allocation of the patients to the various doctors working in the clinic for that working day would provide the following details on a separate list: the patient's name, registration number, identification card number and details as to which doctor's room the patient has been allocated to.

It is standard practice for the clinic that such patients would have a full blood count test done (blood is drawn by the respective nurses) mostly before the patient's consultation with their doctors. Since results of clinical investigations of patients are available online, either the researcher or the assistants would then trace the full blood count results from the

computer. This will be done using the details provided on the daily working list which was mentioned earlier.

For patients with platelet counts of equal to or less than 150×10^9g/L, the researcher or the assistants will then find and tag the patient's clinic card and clinical notes in the various consultation rooms with a red tag. They will also remind the doctor who would be attending to the patient to re-direct the patient to the researcher's booth after the patient's consultation has ended if they suspected that the patient might have dengue fever, and if they were going to give the patient another appointment for outpatient monitoring.

The tag placed with the patient's clinic card also serve as a reminder for the attending doctors to redirect the patient to the researcher's booth after each subsequent follow-up consultation. This will be continued until the attending doctor is satisfied that the patient has completely recovered from the febrile illness either by clinical assessment or laboratory markers.

Thus the initial assessment of these patients and the subsequent follow-up consultations will be done by the various doctors working at the primary health clinic first. The researcher and the trained research assistants see the patients after the clinic consultation with their respective doctors had finished.

Recruitment and consent

Once the patient is redirected to the researcher's booth, either the researcher or one of the assistants will explain to the patient and their accompanying relative or friend regarding the study and provide them with written information regarding the study. If the patient agreed to participate in the study, then a consent form will be completed and signed. Should the patient be less than 18 years of age, then the consent form will have to be signed by the patient's parent or legal guardian.

Randomization

Method of sequence generation

A table of random numbers will be used.

Type of randomization

Simple randomisation will be done, with a 1:1 allocation ratio. Participants are supposed to be distributed equally between the two study groups.

Allocation concealment

No concealment of allocation will be employed.

Implementation of the random sequence

Once consent is obtained, either the researcher or the trained research assistant will then allocate the patient into either one of two groups: the control group or the intervention group. This will be done using the table of random numbers. If the patient gets an odd number, they will be allocated to the control group, if the patient gets an even number, they will then be allocated to the intervention group.

Intervention

Method of intervention received in both the control and intervention groups

All the patients suspected to have dengue fever that will be recruited into the study, regardless of randomization group, will be given the standard care in the form of the patient dengue home care card. This is provided by the Malaysian Ministry of Health, which conforms to the World Health Organization guidelines for dengue 2010[1]. Either the researcher or the trained research assistants will read out to each recruited patient what was written on the card.

The information read out to each recruited patient includes the dengue warning signs, and also the advice on oral fluid intake. This consists of advice on the minimum amount of fluid to drink per day which is at least 5 glasses. There is also advice on what type of fluids one should drink in a day. Patients are advised not to drink plain water only, but to consume barley, fruit juices, milk and isotonic drinks. There is also advice regarding the general self-care measures to be taken by anyone who is suspected to have dengue fever, such as adequate rest, tepid sponging during periods of high fever, the avoidance of steroids during the illness as well as informing patients that antibiotics are not needed.

The amount of time taken by the researcher and the trained research assistants to verbally read out the information on the dengue home care card for the patients is approximately five minutes. This is done in a uniform manner between the principal researcher and the two trained research assistants.

Method of intervention received by the control group only

Apart from this, the control group will be asked their twenty four hour fluid recall. This means that the patient will be asked to give an estimate of how much fluid they have drunk in the twenty four hours prior to the current consultation with the researcher or the trained research assistants.

This estimate will be given by the patients in terms of how many litres or millilitres of fluid they drank, how many glasses (small or large sized glasses or mugs), or how many bottles of drinks they had (500ml size, 1 litre size, or 1.5 litre size), or how many packet drinks they had. Details on what type of fluids is consumed orally will be obtained. This information will be obtained during recruitment of the patient, and on each subsequent follow-up.

One should take note that no fluid chart or cup will be given to the control group participants.

Method of intervention received by the intervention group

The patients assigned to the intervention group will be given a plastic cup of uniform size, along with a twenty four hour fluid chart. These patients will be instructed to use the cup for drinking any form of fluid they wish to consume for the duration of their participation in the study. Verbal instructions will be given on how to fill in the various details required on the fluid chart regarding their oral fluid consumption.

This will be done over the next twenty four hours or until they come for their next follow-up consultation at the clinic. If the next clinic follow-up will be given at the interval of a few days after the preceding consultation, then the researcher or the trained research assistant will provide enough fluid chart forms for the patient to fill until the next consultation.

The patient will be instructed to write down the amount of fluids they drank according to the time that they consumed the fluids in the second column. The patients will be instructed to record the type of fluids they drink in the third column. The patient will only fill the last column if they received any intravenous fluid drip over the next twenty four hours. They are to record the amount of bottles of fluid they receive intravenously.

Blinding

There will be no blinding done for this pilot study. The blinding process is not possible in the intervention and assessment process because the intervention tool as well as the dengue home care card require verbal explanations to be given to participants. Thus, the research team members and the participants will not be blinded in the intervention and assessment process.

Data collection

The demographic, clinical and laboratory data will be collected accordingly. Refer to the Figure 2 for a flow diagram for the data collection process and to appendix 2 for the data collection form.

Follow-up

Duration

All the patients recruited into the study will be followed up until they are clinically better and/or their laboratory markers show improvement as assessed by the respective doctors. Thus, different patients will be followed-up for different lengths of time.

Control group

The researcher or the research assistant will obtain data regarding the twenty four hour fluid recall and the types of fluids consumed for each subsequent follow-up visit.

Intervention group

The researcher or the research assistant will collect the fluid chart given previously and clarify with the patient regarding their entries on the fluid chart. After the patient's consultation with their respective doctors, the researcher or the assistant will give a new fluid chart to the patient if they had been given a another clinic appointment for outpatient monitoring.

Both groups

The following data will be recorded on the data collection form for each subsequent clinic follow-up visit: Their blood investigations, namely their haemoglobin, haematocrit levels, total white cell count, platelets, if dengue serology had been taken, as well as their temperature readings. The participants' management will be handled by their respective doctors. Thus, it is at their discretion whether or not to do a dengue serology test, and the timing of the test. Generally a full blood count test will be ordered for each subsequent clinic visits for outpatient monitoring

Some of the patients have to pay for their own blood test if they were not in government service. However, should the dengue serology results yield a positive IgM result, then the patients can claim the money they had spent for the blood tests. This reimbursement is borne by the Malaysian Government.

Blood tests

Method

The full blood count test will be done using the Sysmex XT-1800 and Sysmex XT-2000 machine at the laboratory situated on the ground floor of the clinic. The platelet count will be measured using the hydrodynamic focusing (DC) detection mechanism, whereas the haematocrit level will be calculated via the red blood cell (RBC) pulse height detection method. Both of these methods will be performed by the Sysmex XT machine.

The dengue serology test will be performed using the Standard Diagnostics Dengue IgM capture ELISA, which is done at the medical virology laboratory in the main situated in the main hospital building. The mean sensitivity of this test is 97.6%, with a 95% confidence interval of 96.8%-98.4%. The mean specificity of this test is also high, which is 90.0%, with a 95% confidence interval of 88%-91.7%[50].

However, no confirmatory tests will be done such as PCR as such tests are very expensive, unavailable in the normal primary healthcare setting, and would not have an impact in the management of these patients. The IgG titres will not done for the participants in this study, even though many of whom have a paired sera sample. This was because this was not the standard practice for the laboratory handling the dengue serology specimens from the primary healthcare clinic.

Tracing

Either the researcher or the trained research assistants will trace the full blood count daily using the online computer system for patient recruitment or follow-up purposes. The results of the dengue serology test will be traced one week after the date of testing. All the information obtained will be recorded on the data collection sheet.

Defaulter tracing

For those who does not turn up for the follow-up with the researcher or the trained research assistants, an attempt will be made to contact the patient via the telephone, with a maximum of three attempts. During the telephone consultation, either the researcher or the trained assistant will attempt to find out the reason why the patient did not come for the follow-up appointment that was given. The patient will then be asked whether they will be amenable to come for follow-up the next day or at the next nearest date when convenient.

If the patient did not come for follow-up as he or she has been admitted to hospital, then details regarding the admission will be obtained. Such details will include which hospital they have been they admitted to, the date of admission, the reason for the admission and who recommended for the admission i.e. was it by medical personnel or did the patient themselves request for the admission. All these details would be entered on the standardized data collection form.

Preliminary outcome measures

The outcomes will be assessed at the end of the two month period of data collection.

Several preliminary outcomes measures will be used to assess the trend of the preliminary results in order to determine the treatment effect size of the fluid chart. The two pilot study groups will be compared in terms of:

1. The hospitalization rates
2. The need for intravenous fluid treatment
3. The severity of haemoconcentration. Four surrogate endpoints were utilized to determine this, and they were as follows:
    a. The mean peak haematocrit
    b. The difference in haematocrit level between the values of clinic consultation 2 – clinic consultation 1.
    c. Those above the local cut off value of haematocrit indicative of plasma leakage.
    d. Haematocrit fluctuation of 20% or more
4. With regards to assessing the severity of the platelet drop, two secondary endpoints were used:
    a. The mean nadir platelet count
    b. The difference in the platelet level between the values of clinic consultation 2 – clinic consultation1.
5. The difference in the amount of oral fluid intake between the two groups.

Estimated sample size

This pilot study will be time driven, as the pre-specified time limit for data collection is two months. The minimum recruited number of participants required for each study group will be 30 patients, giving a minimum number of 60 patients in total. However, the research team strived to recruit as many patients as possible during those two months. This is to determine the actual treatment effect size, and the participant recruitment and retention rates to be used in the sample size calculation for the main evaluation trial.

A prior study has been conducted at the same primary healthcare centre that this pilot study will be performed, assessing outpatient management of dengue illness. This prior study hads a default rate of 16.4% amongst the outpatient suspected dengue cases, which was approximately half of the average default rate of patients attending the general outpatient clinic at University Hospital (33.8%) [51]. However, the current default rate for suspected dengue patients at the primary healthcare clinic might have changed since then.

Statistical analysis

All the data for this pilot study will be expressed either as the mean ± standard deviation or as frequencies and proportions. Differences in data between the control and intervention group will be analysed using the Student's t test for continuous variables. The chi-squared test (χ^2 test) or the Fisher's exact test will be used for categorical variables, whichever is appropriate. A p value < 0.05 is considered as statistically significant. The statistical software utilised for the analysis is SPSS version 16.0 (SPSS Inc. Cary, NC).

Definitions, terminology and assumptions

Definitions

Thrombocytopenia

This is defined as a platelet count of $< 150 \times 10^9$g/L. This is the definition used by another Malaysian study [47].

Severity of haemoconcentration:

Haematocrit fluctuation of 20% or more

This was calculated using the following standard formula:

> Haematocrit fluctuation = (highest haematocrit – population baseline haematocrit) / population baseline haematocrit X 100%

The average population baseline haematocrit value for a 20 year old adult Malaysian male was 0.45 and for the average 20 year old Malaysian female was 0.38. These average population values were obtained and utilised with kind permission from Professor Lucy Lum by personal communication based on her unpublished DENCO study data.

The author will use the average population baseline haematocrit rather than the individual patient's own values because no convalescent sample is routinely done for all the patients enrolled in this pilot study. Moreover, it has been shown by a prior study done in Vietnam which had correctly identified 94% of shocked patients as having capillary leak by utilising the local population mean haematocrit as the baseline for calculating the 20% fluctuation in haematocrit level[43].

Also, the length of patient follow-up varies from patient to patient depending on the attending doctor's assessment of the patient. This secondary endpoint is chosen as a measure for haemoconcentration based on the previous World Health Organization's guidelines for dengue, which included 20% fluctuation in haematocrit as one of the four diagnostic criteria for dengue haemorrhagic fever (DHF).[15] A local Malaysian study also found a fluctuation of 20% haematocrit level to be highly associated with Dengue Haemorrhagic Fever (using the previous World Health Organization classification system)[44].

Above the local cut off value indicative of plasma leakage

This is measured by calculating the percentage of patients achieving and surpassing the local Malaysian population cut off value for haematocrit to suspect plasma leakage. According to a recent study done on the Malaysian population, the cut off value that had been validated for Malaysian males was 0.47, and for the Malaysian females was 0.40[44].

Day of illness

Day 1 of illness is defined as the day of onset of fever. The subsequent day of illness uses day 1 of illness as a reference.

Dengue phase of illness

The dengue phase of illness is based on the day of illness on recruitment into the study, which is as follows:

- The febrile phase is defined as day 1 of illness till day 3 of illness.
- The critical phase is from day 4 of illness to day 6 of illness.
- The recovery phase is defined as from day 7 onwards of illness.

Patient diagnosis

The final diagnosis of the patients who are recruited into the study is defined as follows:

- The patient is defined as having dengue fever if the dengue IgM is positive in either the first sample or the second sample of dengue serology.
- Patients are defined as having probable dengue fever if the dengue IgG is positive regardless of the IgM.
- If both the dengue IgM and IgG are negative, then the patient will be given the diagnosis of other febrile illness.

Body Mass Index (BMI)

In contrast to other infectious diseases, there is evidence to show that severe forms of dengue are more common in well-nourished children. Grade 2 or 3 protein-calorie malnutrition has been shown to be protective against severe dengue vasculopathy[52]. For this reason, BMI is included in the clinical data to be captured during the recruitment of participants into this study.

The Body Mass Index (BMI) is classified using the Malaysian Clinical Practice Guidelines for Obesity[53]:

- BMI < 18.4 was defined as underweight
- BMI of 18.5 – 22.9 was defined as normal
- BMI of 23.0 – 27.4 was defined as pre-obese
- BMI of 27.5 – 34.9 was defined as obesity class I
- BMI of 35.0 – 39.9 was defined as obesity class II
- BMI > 40.0 as obesity class III

Types of fluid

The types of fluid that are consumed by the patients were defined as follows:

- Water – plain water.
- Isotonic drinks – meant either commercially available isotonic drinks or the oral rehydration salts which were made up to be isotonic drinks.
- Juice – meant any type of fruit juice.
- Milk – meant any type of milk.
- Barley – meant either the barley drinks that are commercially available or those that were home-made.
- Caffeinated drinks – meant any drinks containing caffeine, for example coffee, tea or nescafe.
- Nourishing fluids – included the following: alpha lipid drink, Horlicks, vitagen, soup, honey drink, milo drink, and yoghurt drink.
- Semisolids – included the following: oats, and porridge.
- Cordial drinks – consisted of any type of cordial and carbonated drinks.
- Soya drink – meant drinks made from soya bean, either commercially available or home-made
- Home remedies – this included a variety of drinks, including home made liver soup, herb drinks, chloro ‘B’, home-made dates juice, home-made honey and lime drink, home-made ‘assam jawa’ drink.

Amount of fluid in the cups used for the intervention group

Each of the uniform sized cups used in the intervention group contains up to 175 ml of fluids. The principal researcher measures the amount of fluid a cup will contain by filling the cup with water using a syringe to estimate the amount.

Defaulted follow-up and drop out

The term defaulted follow-up means that the patient comes to see the researcher at least once after recruitment into the study. This is not interchangeable with the term drop out, meaning the patient is only seen once by the researcher or the assistants during recruitment, and does not return to see the research team at all after recruitment.

Assumptions

The author has to make several assumptions while conducting this research.

Intravenous fluid drip

If the patient was admitted to hospital after recruitment, it is assumed that the patient would have received intravenous fluid drip.

Conversions used for calculation of total fluid intake

The author has to make several assumptions to ease the calculation of total oral fluid intake, due to the very nature of the data collected from the patients. It is found that patients used a variety of containers of varying shapes and sizes for their daily oral fluid intake.

- For the control group, each glass that the patient reported at home contained 175 ml of fluids, which is equivalent to the amount of fluid contained in the cup used in the intervention group.
- A regular, standard sized mug contains 250 ml of fluids (the principal researcher measured the amount of fluid a mug of such size will contain by filling the mug with water using a syringe to estimate the amount).
- One large cup used by the patient at home is equivalent to one mug, which contains 250ml of water.
- One plastic bag of drinks bought from the hawker stall is equivalent to three cups of water
- One small packet drink contains 250 ml of fluid (as is written on the packet drink itself).
- One can drink contains 325 ml of fluid (as is written on the can itself).
- One small bottle of mineral water contains 500 ml of fluid (as is stated on the bottle label itself).
- One big bottle of drinks contain 1000 ml of fluid (as is stated on the bottle label itself).
- One large bottle of drinks contain 1500 ml of fluid (as is stated on the bottle label itself).
- One jug of water contains 1000 ml of fluid
